# Sensory Processing Challenges in Children with Neurodevelopmental Disorders and Genetic Conditions: An Observational Study

**DOI:** 10.3390/neurosci5030027

**Published:** 2024-09-12

**Authors:** Ekaine Rodríguez-Armendariz, María Vela-Romero, Adrián Galiana

**Affiliations:** 1 Facultad de Ciencias de la Salud, Universidad Europea Miguel de Cervantes (UEMC), 47012 Valladolid, Spain; erodrigueza@uemc.es; 2 Centro de Investigación Micaela Portilla, Universidad del País Vasco (UPV/EHU), 01006 Vitoria, Spain; 3 Conecta Clínica-Centro de Desarrollo Infantil, 13001 Ciudad Real, Spain; direccion@conectaclinica.com; 4 Facultad de Psicología, Ciencias de la Salud y del Comportamiento, Universidad a Distancia de Madrid (UDIMA), 28400 Collado Villalba, Spain

**Keywords:** sensory processing, neurodevelopmental disorders, autism spectrum disorder, attention deficit hyperactivity disorder, learning disorder, developmental disorder, Williams syndrome, 22q11.2 deletion syndrome, pseudohypoparathyroidism, children

## Abstract

Sensory processing challenges are crucial yet often neglected aspects in the care of children with neurodevelopmental disorders and genetic conditions. They represent a key area of interest in neuroscience, as they significantly impact children’s daily functioning and quality of life. This observational study examines these challenges in a group of 614 children, aged 3 to 14 years and 11 months, divided into three groups: 183 with neurodevelopmental disorders (autism spectrum disorder, attention deficit hyperactivity disorder, developmental delays, and learning disorders), 89 with genetic conditions (22q11.2 deletion syndrome, Williams syndrome, and pseudohypoparathyroidism), and 342 controls. Sensory processing was assessed using Sensory Profile 2 (SP2). Results indicated that children with neurodevelopmental disorders and genetic conditions exhibited significant sensory processing difficulties compared to controls. SP2 identified distinct sensory challenges across different sensory systems, varying by diagnosis. Notably, genetic conditions appeared to have a more generalised impact across multiple sensory systems, while neurodevelopmental disorders tended to affect specific systems more narrowly. These findings highlight the importance of early identification and tailored evidence-based interventions to address these specific sensory processing issues. Further research should explore the long-term impact of these interventions in these different populations and their integration into broader therapeutic programmes.

## 1. Introduction

### 1.1. Sensory Integration and Sensory Integration Disorders

Sensory integration (SI) is the neurological process that enables the organisation and interpretation of sensory inputs from the body and the environment, involving sensory systems such as vision, hearing, taste, smell, touch, body-related, and movement-related. This process generates an appropriate adaptive behavioural response. Sensory integration theory, now known as Ayres Sensory Integration^®^ (ASI), focuses on the active and dynamic sensory-motor processes that support movement and interaction within both physical and social environments, thereby promoting development. According to the ASI theoretical framework, the proper functioning of the central nervous system (CNS) is essential for successful sensory integration and processing, which in turn facilitates adaptive responses [1,2,3].

Sensory integration disorders (SIDs) manifest as challenges in performing and participating in daily life. For children, these challenges include difficulties with play, daily activities such as dressing, eating, and personal hygiene, as well as education, social interactions, and sleep [1,4,5]. Following the ASI framework, sensory systems are crucial for the effective integration and processing of sensations during development. Inadequate sensory integration and processing of sensory inputs within the CNS can result in difficulties with sensory reactivity (which can produce problems with sensory modulation) and/or perception (expressed through problems with praxis). In any sensory system, perception fundamentally informs planned action and cognitive processes. Sensory modulation, on the other hand, plays a crucial regulatory role, related to attention, arousal, activity level, and emotion regulation [3]. When these integration issues are present, they can lead to behavioural consequences that significantly impact occupational engagement, thereby affecting a child’s ability to participate fully and effectively in various aspects of daily life.

According to prevalence studies, SIDs may be present in 10–20% of typically developing (TD) children [4,5,6,7]. Studies conducted in Spain report this prevalence as approximately 14% [8], consistent with findings from other countries. However, in conditions affecting neurodevelopment, the prevalence is notably higher, ranging from 40 to 90%, depending on the condition [9]. Given the reported prevalence rates of SIDs in both pathological and non-pathological populations, it is pertinent to advance our understanding of SIDs and explore how they manifest across a broader spectrum of neurodevelopmental disorders and genetic conditions associated with CNS alterations.

Understanding how SIDs may differentially affect sensory systems depending on the specific pathological condition could assist professionals in comprehending their impact on a patient’s daily life functioning and support evidence-based clinical decision-making.

### 1.2. Sensory Integration Disorders and Neurodevelopmental Disorders

Alterations in brain sensory processing structures may result from disrupted neurodevelopmental processes. Neuroimaging studies have revealed that children with SIDs exhibit altered white matter microstructure, affecting both the posterior cerebral tracts and cerebellar networks responsible for sensory processing and integration. This disruption in microstructural integrity has been correlated with atypical sensory behaviour [10,11] explaining, at least in part, SID manifestations in neurodevelopmental disorders.

As previously stated, SIDs have been extensively studied in the context of autism spectrum disorder (ASD). This neurodevelopmental disorder is characterised by persistent deficits in communication and social interaction, alongside restrictive and repetitive patterns of behaviour, interests, or activities. Additionally, individuals with ASD can exhibit a wide variability in the severity and presentation of symptoms, ranging from significant delays in language development and social skills to superior intellectual abilities in specific areas [12]. Sensory difficulties are present in up to 90% of ASD cases, making them one of the most impactful issues affecting behaviour and participation [13].

Another neurodevelopmental disorder that has been studied in the context of SIDs, although less extensively than ASD, is attention deficit hyperactivity disorder (ADHD). This disorder is characterised by a persistent pattern of inattention and/or hyperactivity–impulsivity that interferes with the individual’s normal functioning or development. Symptoms of inattention include difficulties in maintaining focus, following instructions, and completing tasks, while symptoms of hyperactivity–impulsivity may manifest as constant restlessness, frequent interruptions, and difficulty waiting for one’s turn [12]. Studies suggest that sensory-related difficulties associated with ADHD are common and negatively impact stress levels and promotes disruptive behaviours [14,15]. However, as noted, further research is necessary to advance our understanding of these relationships.

The study related to sensory processing initially focused on children with learning difficulties [16]. Therefore, it is considered relevant to include children with learning disorders (LDs) or developmental delay (DD) in this study, due their school achievement difficulties.

Regarding DD, it is characterised by significant delays across multiple developmental areas, including motor, cognitive, social, and language skills. Children with DD typically exhibit slower progress compared to their peers and may face considerable challenges in achieving typical developmental milestones such as crawling, walking, and speaking [12]. Children with DD may experience sensory integration issues that are usually linked with DD etiological factors related with pre-, peri-, and postnatal issues such as exposure to maternal chronic diseases and substance abuse, premature birth, or environmental influences (poor nutrition, a lack of stimulation, and others) [17].

Living with LDs, according to DSM-5, involves difficulties in specific academic skills such as reading, writing, and mathematics. These disorders are marked by performance that is significantly below what is expected for the child’s chronological age and educational level, adversely affecting their academic performance and self-esteem. Difficulties in sensory integration can lead to a poor perception of the environment, affecting the way a child interprets and responds to academic information. For example, difficulties in processing visual information and movement can negatively impact reading, writing, and/or mathematics [18]. However, little is known about which specific sensory modalities may be altered in specific LDs [19].

### 1.3. Sensory Integration Disorders in Genetic Conditions

A variety of genetic conditions are known to affect brain development and, consequently, might potentially exhibit behaviours related to SIDs, as they impact areas involved in sensory processing and the perceptual integration of inputs. This is the case of Williams syndrome (WS), 22q11.2 deletion syndrome (22qDS) and pseudohypoparathyroidism (PHP). Although some previous research indicates sensory processing alterations in WS [20], this area remains largely unexplored in 22qDS and PHP.

The condition 22qDS, caused by a deletion in the q11.2 region of chromosome 22, is associated with significant brain abnormalities, along with cardiac anomalies, cleft palate, immune deficiencies, cognitive difficulties, and an increased risk of psychiatric disorders such as schizophrenia [21]. Common structural alterations include hypoplasia of the corpus callosum, which impacts interhemispheric communication, and anomalies in the thalamus, affecting the relay of sensory and motor information. Additionally, structural changes in the cortical brain regions, such as variations in cortical thickness, have been observed. These structural abnormalities are linked to disruptions in neural networks and can contribute to deficits in cognitive and emotional functions, impacting development and behaviour in individuals with the syndrome [22].

WS is caused by a microdeletion in the chromosomal region 7q11.23. It is characterised by a distinctive cognitive and behavioural profile, including strong social and verbal skills, accompanied by anxiety and attention problems. Additionally, individuals with WS may present with cardiovascular anomalies, hypercalcemia in infancy, and a distinctive facial phenotype [23]. MRI studies reveal reduced brain size and a more pronounced loss of white matter compared to grey matter in WS. The posterior brain regions are notably more affected, with reduced grey matter density observed in the superior parietal lobe and hypofunction near the intraparietal sulcus, areas associated with multisensory integration and perception [24].

PHP is a rare genetic disorder caused by mutations in the *GNAS* gene, which affects a complex network of signalling pathways that ultimately influence various cellular functions by regulating hormone activity. PHP is characterised by the body’s resistance to parathyroid hormone, leading to low calcium levels and high phosphate levels in the blood. Despite normal or elevated PTH levels, the body’s tissues do not respond appropriately, resulting in symptoms similar to hypoparathyroidism, such as muscle cramps, tetany, and neurological issues [25]. Additionally, extensive brain calcifications can occur in areas such as the bilateral basal ganglia, cerebellum, thalamus, and cerebral cortex, potentially leading to neurological and cognitive impairments [26].

### 1.4. Objective

Given these precedents, it is hypothesised that, similar to disorders extensively studied in the context of SIDs, such as ASD, other neurodevelopmental disorders and genetic conditions affecting brain development might exhibit differential sensory processing alterations.

Thus, the main goal of this study was to contribute to the knowledge of SIDs not only in well-studied conditions such as ASD, but in other, less studied but prevalent neurodevelopmental disorders like ADHD, LDs, or DD. Furthermore, this study makes an original contribution by exploring a triad of genetic conditions known to affect central nervous system development, with the aim of investigating whether, and in what manner, they impact sensory processing. These conditions are 22qDS, WS, and PHP.

## 2. Materials and Methods

### 2.1. Participants

Children aged between 3 and 14 years and 11 months were recruited to participate in the study. Participants were assigned into different groups based on primary developmental or genetic condition, as follows: TD children, children diagnosed with a neurodevelopmental disorder (ASD, ADHD, DD, and LDs), and children with genetic conditions (22qDS, WS, and PHP).

The TD group was recruited from a sample of children without any developmental disorders or medical conditions from six public primary schools across six different cities in central-southern Spain [8]. Patients with neurodevelopmental disorders were recruited from families who routinely and voluntarily attend a paediatric clinic specialising in neurodevelopmental disorders. Participants with genetic conditions were recruited from various national associations of patients with the aforementioned genetic disorders.

Participants were selected based on specific inclusion and exclusion criteria to ensure the validity of the study. For inclusion in the normative group, participants needed to be within the specified age range (3 years to 14 years and 11 months), and have a complete sensory profile. Exclusion criteria for the normative group included any diagnosed or suspected medical or developmental conditions, incomplete sensory profiles, and a lack of informed consent. For the diagnostic groups, participants were required to be within the same age range and have a complete sensory profile. Diagnoses of neurodevelopmental disorders or genetic conditions were previously assigned by clinicians from the Spanish National Health System, based on DSM-5 criteria and genetic analysis, respectively. The exclusion criteria for the diagnostic group were the presence of comorbid disorders, incomplete sensory profiles, and the lack of informed consent.

Participation in the study was voluntary. All participants and their legal guardians were thoroughly informed about the objectives of the project, as well as its benefits and costs, and informed consent was obtained in all cases.

### 2.2. Sensory Processing Measure

This study aims to explore sensory processing characteristics of children with different neurodevelopmental disorders and genetic conditions, compared to TD children. A variety of tools are available to researchers and clinicians seeking to conduct both screenings or comprehensive assessments of behaviours related to sensory processing difficulties [27,28,29,30].

Typically, sensory processing screening tools involve short questionnaires that assess the frequency of behaviours related to impaired sensory processing (Short Sensory Profile [31] or Sensory Processing Measure [32]). As screening tools, the clinical potential of these questionnaires is limited. However, there are extended versions that analyse sensory reactivity processing in greater depth and provide information related to each sensory system and their behavioural impact, such as Sensory Processing Measure, Second Edition (SPM−2) [28] and Sensory Profile 2 (SP2) [27]. All these questionnaires are parent-reported.

SPM−2 and SP2 were originally developed in English, with translations available for Spanish-speaking populations. Furthermore, SP2 includes normative data for the Spanish population. In the present study, the extended Spanish-adapted version of the SP2 questionnaire was used [27]. SP2 is the updated version of the original Sensory Profile (SP) [31,33] that was developed for assessing sensory processing alterations. Owing to its simplicity and reliability, the first short version of SP has been commonly used to screen the prevalence of sensory processing issues [4,5,6,7]; however, current studies also include the extended version for this purpose, aiming to provide a more in-depth analysis [8,34]. The short and extended versions of Sensory Profile are designed to be completed by parents or caregivers for children aged between 3 and 14 years and 11 months, and can be scored manually according to the instructions in the manual or through the online correction platform provided by the publisher.

SP2 consists of 86 items divided into two main sections: the sensory section and the behavioural section. The sensory section assesses auditory, visual, tactile, movement, body position, and oral sensory processing, while the behavioural section addresses conduct, social-emotional responses, and attentional responses related to sensory processing. The combination of responses from items in both sections provides an interpretation of the scores, typically presented in four quadrants: seeking, avoiding, sensitivity, and registration. However, a recent study concludes that there is moderate to strong convergent validity only for the sensory section analysed in the Spanish versions of Sensory Profile and Sensory Processing Measure for children with sensory integration issues [35], and not for the behavioural section or quadrants.

Considering these recent findings and the objective of the study, the analysis focused on the sensory section.

The Spanish-adapted version of SP2 has been widely used in clinical contexts for sensory processing disorders among Spanish-speaking populations, primarily as a guide to comorbid manifestations in neurodevelopmental disorders such as autism and ADHD [36,37]. However, in Spain, only a few studies have been conducted to explore this topic [8,38,39,40].

For each of the measures provided by SP2, the reliability coefficients for the Spanish normative sample range from adequate (0.74) to excellent (0.87). Furthermore, the test–retest reliability coefficients range from 0.87 to 0.97, and the inter-rater reliability coefficients range from 0.73 to 0.89 [27].

Additionally, validity studies demonstrate a clear correlation with other similar instruments, such as SP, Sensory Profile School Companion (SPSC), Behavioural Assessment System for Children−2 (BASC−2), and Vineland Adaptive Behavior Scales−2 (VINELAND−2), among others [27].

### 2.3. Procedure

The researchers recruited participants by contacting public schools for the TD group, collaborating with a specialised paediatric clinic to enrol children with neurodevelopmental disorders, and working with national patient associations to form groups for genetic conditions.

In this cross-sectional study, informed consent and written instructions were provided to all participants, who received information on correctly completing the SP2 questionnaire, along with a summary of the study’s objectives and theoretical framework. A telephone number and email address were also provided to enable contact with the research team for any further questions at any stage of the project.

All documentation was collected and reviewed by the researchers. The questionnaires were scored online.

All participants in this study provided informed consent. This research is part of the EPIDIS (Epidemiology of Sensory Integration Disorder) project, which was previously authorised by the Official College of Occupational Therapists of Castilla-La Mancha (COFTO-CLM) under reference COFTOCLM-IV−2017. The COFTO’s research committee reviewed the procedures to ensure compliance with bioethical standards for human research in accordance with current ethical guidelines.

### 2.4. Statistical Analysis

Since the data did not meet normality assumptions, statistical significance was assessed using non-parametric analysis. Differences among groups for each sensory section were evaluated using a Kruskal–Wallis analysis of variance, followed by the Benjamini, Krieger, and Yekutieli procedure to correct for false discovery rate. The effect size of each comparison was calculated using Cliff’s delta statistic, with *p*-values < 0.05 considered significant. GraphPad Prism v8.0.1 was used for performing calculations and generating graphs.

## 3. Results

Table 1 shows the demographic results for each group including the number of participants, sex, and age. The total sample consisted of Caucasian boys and girls, including 342 participants with typical development, 183 with neurodevelopmental disorders (35 with ADHD, 47 with ASD, 36 with DD, and 65 with LDs). Additionally, 89 participants with genetic conditions were recruited (35 with 22qSD, 40 with WS, and 14 with PHP).

For each group, the proportion of boys and girls was statistically equivalent (*p*-values greater than 0.05 in a z-test proportion contrast), except in the ADHD, ASD, DD, and LD groups, where there was a significantly higher representation of male participants (Table 1).

**Figure 1:**
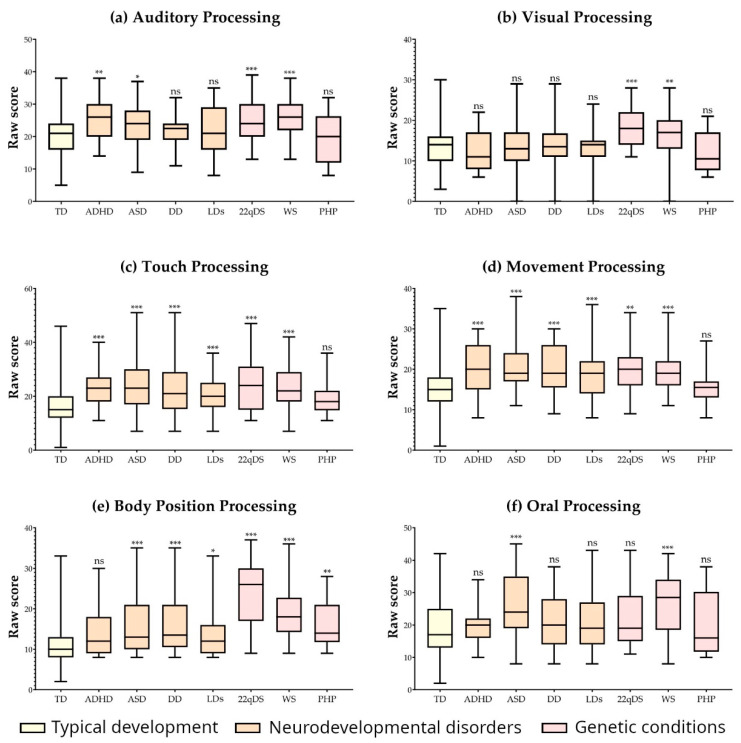
Mean raw scores from Sensory Profile 2 (SP2) across different groups for each sensory section. (**a**) Auditory processing; (**b**) visual processing; (**c**) touch processing; (**d**) movement processing; (**e**) body position processing; (**f**) oral processing. *Note:* TD: typical development; ADHD: attention deficit hyperactivity disorder; ASD: autism spectrum disorder; DD: developmental delay; LDs: learning disorders; 22qDS: 22q11.2 deletion syndrome; WS: Williams syndrome; PHP: pseudohypoparathyroidism; ns: not significant (*p* > 0.05); *: *p* ≤ 0.05; **: *p* ≤ 0.01; ***: *p* ≤ 0.001.

Table 2 and Figure 1 summarise the results corresponding to the sections of SP2 that evaluate the frequency of behaviours, which are associated with disturbances in the following sensory processing systems: auditory, visual, tactile, movement (related to vestibular functions), body position (associated with proprioception), and oral. Means and standard deviations for each sensory processing system and group are shown. Additionally, this table includes the H statistic values from the Kruskal–Wallis test for group comparisons in each sensory section. Finally, *p*-values of Dunn’s post hoc test, comparing each group’s results with those of the TD group, are presented in both Table 2 and Figure 1.

Table 2 and Figure 1a–f indicate that, compared to the TD group, auditory processing was significantly impaired in the ADHD, ASD, and 22qDS groups. In visual processing, the 22qDS group showed significant differences compared to the TD group. All conditions, except PHP, exhibited impairments in tactile and movement/vestibular processing. All conditions except ADHD showed altered body position/proprioceptive processing, while the ASD group had impairments in oral processing. Notably, the WS group showed disturbances across all systems, being the only group with this characteristic.

## 4. Discussion

The effective processing of sensory information from the body and the environment enables children’s active participation at home, in school, and within the community. SI supports development and becomes increasingly complex as children grow, adapting to their changing needs and environments [1,3,41]. This adaptive process is essential for fostering appropriate responses to sensory stimuli, which in turn influences their ability to engage in daily activities and social interactions effectively.

**Figure 2:**
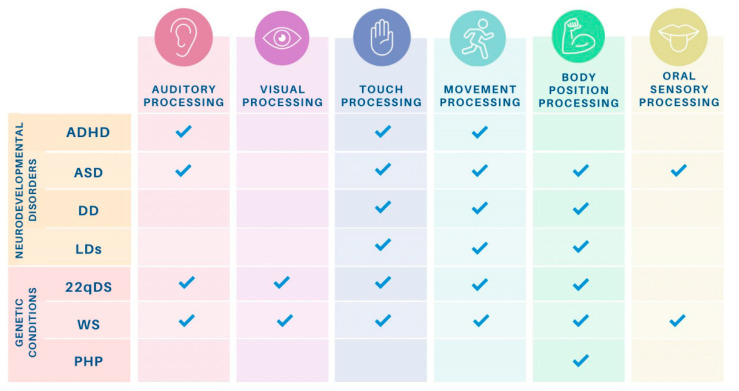
Visual correlation matrix for the sensory processing characteristics in the sample of studied neurodevelopmental disorders and genetic conditions. *Note:* ADHD: attention deficit hyperactivity disorder; ASD: autism spectrum disorder; DD: developmental delay; LDs: learning disorders; 22qDS: 22q11.2 deletion syndrome; WS: Williams syndrome; PHP: pseudohypoparathyroidism.

The scientific literature provides several relevant findings related to sensory processing in both neurodevelopmental disorders and genetic conditions, as will be discussed below. This study and its results significantly contribute to describing a common characterisation of SID, thereby enhancing the existing body of literature. By doing so, it promotes a deeper understanding of SIDs and provides a framework for improved early identification and intervention for children and their families. To provide a graphical summary of the main findings, a visual correlation matrix including auditory processing, visual processing, touch processing, movement processing, body position processing, and oral sensory processing is presented in Figure 2.

### 4.1. Neurodevelopmental Disorders

Neurodevelopmental disorders may exhibit variability in sensory difficulties; however, they share common characteristics such as deficits in development and performance, which are evident from an early age and have a neurobiological basis [12].

In 2013, with the publication of DSM-5, difficulties in sensory modulation, described as hyper- or hypo-reactivity to sensory inputs, were introduced as diagnostic criteria for ASD [12], highlighting the relevance of SID-related behaviours. There is a growing body of evidence regarding modulation difficulties in autism, that indicate an association between sensory issues and the core characteristics of the disorder [42,43,44]. Studies using parent-reported questionnaires primarily describe a general impact on all sensory systems, affecting overall functioning [45,46]. However, the results suggest that, at least in the ASD sample of the present study, the visual system appears to be the least impacted sensory processing system. Despite the previously reported presence of visuomotor and other capabilities related to visual processing in autism [47], the results are consistent with the widely accepted notion that the visual system may represent a potential strength for these children, rather than a weakness; furthermore, the visual system supports a variety of the therapeutic interventions designed for learning and communication skills in ASD [48,49]. Given the known complexity and variability of autism, and considering the reported difficulties in sensory processing across a wide range of sensory systems, information provided by visual stimuli may offer valuable support for therapeutic intervention, as this processing system appears to be less affected than others.

ADHD is one of the most prevalent neurodevelopmental disorders in childhood and adolescence [50]. Given the high rates and impact of this condition on personal and academic life [51], researchers have sought to explore whether sensory processing might play a role in ADHD difficulties. Several studies using SP2 have identified differences in sensory processing across all sensory systems assessed [34,45]. However, another study employing SPM [14] found alterations in the touch, vision, balance, hearing, and visual systems, without finding relevant results on body-related information, similarly to the present study. It is important to indicate that SPM did not include oral processing scores, so it is not possible to compare the results in this area. A study conducted in Spain in 2020, which administered the short form of SP2, reported results across the Dunn model quadrants, providing general information related to overall systems, but did not detail results by specific sensory systems [38]. The present study improves this aspect, providing detailed information by sensory systems, describing difficulties in the auditory, touch, and movement-related systems, without identifying alterations in other systems.

Learning disorders were addressed in Ayres’ pioneering research on sensory processing [16]. SI theory primarily focuses on proximal senses (tactile, proprioceptive, and vestibular) and examines their contributions to occupational performance [1,52]. It is noteworthy that the results for DD and LDs—disorders that originally inspired the development of sensory integration theory—are precisely those initially identified in the participants. Existing research highlights difficulties with hyper-reactivity in DD [53], aligning with the initial SI theoretical hypotheses. Through various questionnaires, other studies confirm sensory processing difficulties for DD and LDs that may also include auditory processing in some LD samples [19,46].

Regarding touch processing, all the neurodevelopmental disorders studied exhibited significant difficulties in this sensory modality, as expected, since the poor modulation of tactile sensations is a well-recognised common feature of neurodevelopmental disorders. In Ayres’ early studies, she identified over-reactivity to tactile input, known as “tactile defensiveness”, as being associated with behavioural hyperactivity and distractibility [54]. Furthermore, Schaaf et al. [55] reported that children with sensory hyperreactivity exhibit inadequate parasympathetic activation, evidenced by lower vagal tone, when compared to controls. The researchers suggested that this finding indicates an autonomic imbalance, with an increased parasympathetic response needed to regulate the sympathetic system and the resulting reactivity to sensory stimuli.

The results have also highlighted auditory processing difficulties in ASD and ADHD, in accordance with previous studies that link these difficulties to poor academic performance and impaired social functioning [56,57]. Auditory hypo-reactivity refers to an attenuated response to auditory stimuli, where individuals may not react as strongly to sounds that typically elicit a response in others. This reduced response can lead to a diminished awareness of or engagement with environmental sounds. Conversely, auditory hyper-reactivity involves an exaggerated response to auditory stimuli, where individuals may perceive sounds as excessively loud or intrusive, leading to heightened discomfort or distress. Both auditory hypo-reactivity and hyper-reactivity are well-documented features of ASD [58,59,60]. Regarding ADHD, although there is some evidence linking auditory processing alterations [61], hypo-reactivity seems to be associated with inattention and impaired concentration [62]. However, auditory processing difficulties remain less established in ADHD than ASD and require further research.

### 4.2. Genetic Conditions

Research on genetic conditions impacting brain development reveals a variety of issues related with developmental and cognitive issues affecting learning and social functioning [21,23]. These difficulties, attributed to genetically linked alterations in brain development, suggest that sensory processing may also be disrupted, as the maturation of sensory processing areas and the integrity of their neural circuitry develop during the early years of life. However, the study of sensory processing in genetic conditions is still poorly understood. WS is one of the genetic syndromes that have received some attention in this regard, whereas research on 22qDS is nearly anecdotal and virtually non-existent for PHP.

Studies conducted on children with WS using SP provide information highlighting a general impact on all sensory processing systems [63,64]. These findings are consistent with the results of the present study, which also indicate an impact across all sensory systems. Despite the general sensory processing alterations reported in WS, it is encouraging to note that individuals with this syndrome appear to experience some spontaneous yet significant improvements in sensory processing over time [64]. Additionally, in cases where WS-associated difficulties are minimal, sensory processing shows less impairment, particularly with regard to auditory processing [64]. Finally, it has been observed that the sensory processing characteristics of WS are similar to those of ASD, not only in terms of sensory modalities but also in their functional impact, making it challenging to distinguish between these conditions when considering sensory issues alone [20]. The similarities between the sensory characteristics of ASD and WS highlight the potential value of including sensory modulation measures for WS, as was done for ASD in DSM-5 [12].

Specific research on sensory processing in PHP and 22qDS is limited. For PHP, no relevant studies have been identified, while for 22qDS, there is only one study focusing on sleep problems that mentions difficulties in sensory processing, although it does not provide quantitative data or measurements [65].

To our knowledge, this is the first research report focusing on sensory processing issues in 22qDS and PHP. As previously indicated, structural brain abnormalities have been described in these two genetic conditions: generalised increased cortical thickness and thalamic and interhemispheric communication abnormalities in 22qDS, and calcifications in cortical, subcortical, and cerebellar areas for PHP [22,26]. Regarding the results for 22qDS, sensory processing challenges were similar to those observed in WS, except for oral processing, indicating alterations across nearly all studied systems. In contrast, PHP was primarily associated with difficulties related to body position and proprioception, which implies challenges in processing motor control and information about joint position, muscle tension, and balance. These findings are consistent with the structural brain alterations described for these conditions. In 22qDS, increased cortical thickness and disturbed thalamic and interhemispheric communication networks may be linked to dysfunctional processing in the cortical primary and association areas, affecting sensory perception and integration stages. Conversely, in PHP, the calcification of subcortical structures such as the basal ganglia, the thalamus, and cerebellar structures, would likely be involved in both motor and sensory integration signalling, leading to proprioceptive difficulties. However, these hypothetical associations require further exploration and should be considered in future research.

Currently, the study of sensory processing challenges in the context of genetic conditions that affect brain development has only just begun. In consequence, substantial progress is still needed. Identifying these characteristics facilitates the development of specific intervention strategies and enhances the understanding of the interrelationships between different genetic conditions and their sensory processing-related behavioural consequences, thus providing a foundation for future research and clinical practice.

### 4.3. Limitations and Future Research

This study has a number of limitations. First, the sample size for both neurodevelopmental disorders and genetic conditions was relatively small. A larger and more representative sample, encompassing both an increased number of participants and a balanced gender distribution, would enhance the applicability of the findings. Additionally, expanding the age ranges and including diverse ethnic and cultural backgrounds would provide a more comprehensive understanding of sensory processing characteristics across different developmental stages.

Furthermore, the variety of existing tools to assess sensory processing in children, including both reactivity and perception aspects, is well documented in the literature [1,27,28,29,30,31,32]. However, discrepancies in how results are reported can hinder consensus and clarity in the findings. The data in this study are primarily based on parent-reported information, which largely pertains to aspects of modulation. A comprehensive study of sensory processing would benefit from including measures aligned with SI theory to assess perception, as has been done in ASD populations [66], along with other evaluation tools that could link sensory characterisation to the behavioural manifestations reported by families and the potential challenges in occupational engagement. Additionally, while there is research supporting the altered anatomical substrate in SIDs, it would be valuable to complement observational and performance studies by analysing the underlying neurobiological substrate and brain functioning.

## 5. Conclusions

There is a noticeable alteration in the processing of sensory stimuli in the neurodevelopmental disorders and genetic conditions studied.

For well-studied populations such as ASD and ADHD, the characterisation of sensory processing challenges aligns with the existing literature. Moreover, DD and LDs show results consistent with theoretical hypotheses and current knowledge regarding sensory processing difficulties.

For populations that have not yet been extensively studied, such as the genetic conditions WS, 22qDS, and PHP, this study provides an initial characterisation, revealing sensory processing difficulties that are consistent with neuroimaging findings of brain structural alterations.

Understanding how sensory stimuli are processed in different populations is crucial for tailoring interactions, environmental characteristics, and performance expectations.

This study achieves its initial objectives and paves the way for future research aimed at deepening the knowledge and description of sensory processing characteristics related to neurodevelopmental disorders and genetic conditions in childhood.

## Figures and Tables

**Table 1:** Demographics.

Group		Total	Boys	Girls	z-Value	*p*	
TD	N	342	169	173	−0.22	0.829	(ns)
	%	100	49.42	50.58			
	Age (mean)	7.18	7.14	7.21	−0.02	0.985	(ns)
	Age (sd)	2.19	2.06	2.31			
ADHD	N	35	33	2	2.43	0.015	*
	%	100	94.29	5.71			
	Age (mean)	8.34	8.15	11.50	−0.74	0.457	(ns)
	Age (sd)	2.71	2.68	0.50			
ASD	N	47	37	10	3.22	0.001	**
	%	100	78.72	21.28			
	Age (mean)	6.00	5.51	7.80	−0.62	0.537	(ns)
	Age (sd)	3.64	2.75	5.49			
DD	N	36	28	8	2.77	0.006	**
	%	100	77.78	22.22			
	Age (mean)	3.56	3.43	4.00	−0.21	0.834	(ns)
	Age (sd)	1.40	0.98	2.29			
LDs	N	65	46	19	3.05	0.002	**
	%	100	70.77	29.23			
	Age (mean)	6.48	6.57	6.26	0.08	0.933	(ns)
	Age (sd)	3.11	3.25	2.71			
22qDS	N	35	15	20	−0.84	0.403	(ns)
	%	100	42.86	57.14			
	Age (mean)	9.31	8.67	9.80	−0.26	0.792	(ns)
	Age (sd)	5.27	4.09	5.95			
WS	N	40	25	15	1.53	0.126	(ns)
	%	100	62.50	37.50			
	Age (mean)	7.68	7.80	7.47	0.09	0.932	(ns)
	Age (sd)	3.86	3.76	4.00			
PHP	N	14	8	6	0.53	0.597	(ns)
	%	100	57.14	42.86			
	Age (mean)	9.21	9.25	9.17	0.02	0.985	(ns)
	Age (sd)	3.84	3.99	3.62			

*Note:* TD: typical development; ADHD: attention deficit hyperactivity disorder; ASD: autism spectrum disorder; DD: developmental delay; LDs: learning disorders; 22qDS: 22q11.2 deletion syndrome; WS: Williams syndrome; PHP: pseudohypoparathyroidism; sd: standard deviation; *p*: *p*-value; ns: not significant (*p* > 0.05); *: *p* ≤ 0.05; **: *p* ≤ 0.01.

**Table 2:** Results for SP2 sensory sections in the different groups.

	SP2 Sensory Sections											
	Auditory processing		Visual processing		Touch processing		Movement processing		Body position processing		Oral sensory processing	
Group	mean (sd)	*p/d_Cliff_* (vs. TD)	mean (sd)	*p/d_Clif_* (vs. TD)	mean (sd)	*p/d_Clif_* (vs. TD)	mean (sd)	*p/d_Clif_* (vs. TD)	mean (sd)	*p/d_Clif_* (vs. TD)	mean (sd)	*p/d_Clif_* (vs. TD)
TD	19.94 (6.69)	-	13.51 (4.34)	-	17.05 (6.48)	-	15.82 (5.67)	-	11.09 (4.25)	-	19.61 (7.80)	-
ADHD	24.54 (6.12)	0.001/−0.382	11.94 (4.76)	0.035/0.158	22.91 (6.97)	<0.001/−0.507	20.48 (6.36)	<0.001/−0.462	13.65 (5.74)	0.013/−0.377	19.65 (5.07)	0.474/−0.14
ASD	24.10 (6.55)	0.002/−0.318	13.42 (4.97)	0.799/−0.034	23.78 (8.64)	<0.001/−0.524	20.38 (5.66)	<0.001/−0.514	15.36 (7.32)	<0.001/−0.463	25.34 (10.05)	<0.001/−0.367
DD	21.30 (5.56)	0.342/−0.156	13.94 (5.95)	0.976/−0.054	23.33 (9.35)	<0.001/−0.493	20.08 (5.74)	<0.001/−0.462	16.83 (7.79)	<0.001/−0.569	21.41 (7.98)	0.160/−0.188
LDs	22.00 (7.41)	0.104/−0.154	12.96 (0.99)	0.389/0.012	20.29 (6.48)	<0.001/−0.358	19.16 (6.26)	<0.001/−0.371	13.43 (5.47)	0.001/−0.373	21.16 (8.08)	0.146/0.154
22qDS	25.51 (6.75)	<0.001/−0.432	18.22 (4.67)	<0.001/−0.566	24.42 (9.41)	<0.001/−0.499	19.60 (6.12)	<0.001/−0.414	23.80 (8.04)	<0.001/−0.837	22.60 (8.94)	0.068/−0.228
WS	26.30 (6.25)	<0.001/−0.512	16.60 (5.72)	<0.001/−0.407	23.02 (7.24)	<0.001/−0.528	19.63 (5.11)	<0.001/−0.463	19.07 (5.84)	<0.001/−0.808	26.77 (9.07)	<0.001/−0.462
PHP	19.64 (7.35)	0.765/0.014	12.00 (4.72)	0.225/0.135	19.14 (6.43)	0.229/−0.282	15.35 (4.95)	0.764/−0.028	16.35 (5.66)	<0.001/−0.647	19.92 (9.16)	0.817/−0.015

*Note:* Statistical significance was assessed using a Kruskal–Wallis one-way analysis of variance, followed by a two-stage linear step-up procedure by Benjamini, Krieger, and Yekutieli to correct for multiple testing SP2: Sensory Profile 2; sd: standard deviation; *p*: *p*-value; *d_Cliff_*: Cliff’s delta effect size; TD: typical development; ADHD: attention deficit hyperactivity disorder; ASD: autism spectrum disorder; DD: developmental delay; LDs: learning disorders; 22qDS: 22q11.2 deletion syndrome; WS: Williams syndrome; PHP: pseudohypoparathyroidism.

## Data Availability

Data are unavailable due to privacy and legal restrictions according to the Organic Law on Data Protection.
